# Oseltamivir-Resistant Influenza Virus A (H1N1), Europe, 2007–08 Season

**DOI:** 10.3201/eid1504.081280

**Published:** 2009-04

**Authors:** Adam Meijer, Angie Lackenby, Olav Hungnes, Bruno Lina, Sylvie van der Werf, Brunhilde Schweiger, Matthias Opp, John Paget, Jan van de Kassteele, Alan Hay, Maria Zambon, on behalf of the European Influenza Surveillance Scheme

**Affiliations:** Netherlands Institute for Health Services Research, Utrecht, the Netherlands (A. Meijer, J. Paget); National Institute for Public Health and the Environment, Bilthoven, the Netherlands (A. Meijer, J. van de Kassteele); European Surveillance Network for Vigilance against Viral Resistance (A. Lackenby, B. Lina, S. van der Werf, A. Hay, M. Zambon); Health Protection Agency, London, UK (A. Lackenby, M. Zambon); Norwegian Institute of Public Health, Oslo, Norway (O. Hungnes); Centre National de Référence des Virus Influenza (Région Sud), Lyon, France (B. Lina); Centre National de Référence des Virus Influenza (Région Nord), Paris, France (S. van der Werf); Robert Koch Institute, Berlin, Germany (B. Schweiger); Laboratoire National de Santé, Luxembourg, Luxembourg (M. Opp); World Health Organization Collaborating Centre Medical Research Council/National Institute of Medical Research, London (A. Hay)

**Keywords:** viruses, antimicrobial resistance, influenza A virus, H1N1, antiviral agents, oseltamivir, epidemiology, surveillance, Europe, research

## Abstract

In Europe, the 2007–08 winter season was dominated by influenza virus A (H1N1) circulation through week 7, followed by influenza B virus from week 8 onward. Oseltamivir-resistant influenza viruses A (H1N1) (ORVs) with H275Y mutation in the neuraminidase emerged independently of drug use. By country, the proportion of ORVs ranged from 0% to 68%, with the highest proportion in Norway. The average weighted prevalence of ORVs across Europe increased gradually over time, from near 0 in week 40 of 2007 to 56% in week 19 of 2008 (mean 20%). Neuraminidase genes of ORVs possessing the H275Y substitution formed a homogeneous subgroup closely related to, but distinguishable from, those of oseltamivir-sensitive influenza viruses A (H1N1). Minor variants of ORVs emerged independently, indicating multiclonal ORVs. Overall, the clinical effect of ORVs in Europe, measured by influenza-like illness or acute respiratory infection, was unremarkable and consistent with normal seasonal activity.

## Abstract 2

A high level of virus circulation and introduction of an antigenic drift variant in a susceptible population contributed to the spread of resistant virus.

Infection with influenza viruses A (H1N1), A (H3N2), or B causes substantial human illness and excess deaths each year (*1*,*2*). Vaccination against seasonal influenza is the key control measure used in Europe to minimize illness and death. Antigenic mismatch between vaccine components and circulating viruses occurs every few years, requiring reformulation of the vaccine (*1*). In addition, suboptimal immunization in patient groups for which vaccine is recommended provides the rationale for use of antiviral drugs in the prophylaxis and treatment of influenza. M2 ion channel inhibitors (M2Is), amantadine and rimantadine, have been available since 1964, but adverse effects, rapid development of resistance, and lack of activity against influenza B have limited their usefulness (*3*). The introduction of neuraminidase inhibitors (NAIs), oral oseltamivir and inhaled zanamivir, which are active against both influenza type A and B viruses, was a major breakthrough in treatment and prophylaxis of influenza using antiviral drugs (*4*). However, prescription data indicate that they are not widely used in Europe (Figure 1); by contrast, in Japan during the 2003–04 season alone, ≈6 million NAI treatment courses were prescribed (*5*).

**Figure 1:**
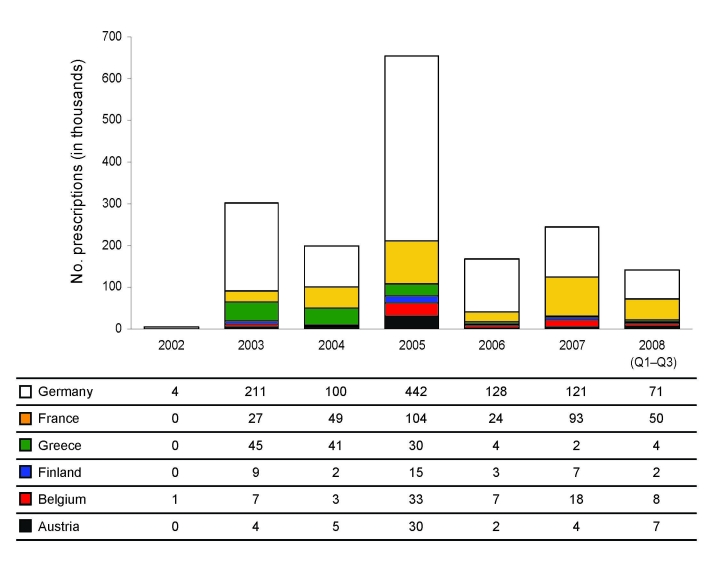
Prescription data of oseltamivir treatment courses for Western Europe (in thousands); 12 months of data for each year 2002–2007 and through September for 2008. Data from the United Kingdom, the Netherlands, Switzerland, and Portugal are excluded because of negligible values. Data provided by IMS Health (www.imshealth.com), London, UK.

Before the introduction of NAIs in 1999, and until 2007, <1% of viruses tested from unselected surveillance studies in a number of countries demonstrated natural resistance to NAIs (*5*–*9*). Limited development of resistance to oseltamivir has been observed in persons treated, with little evidence of onward transmission of resistant viruses (*10*), although low-level transmission of resistant variants cannot be discounted (*11*). However, oseltamivir-resistant viruses emerged in 18% (9/50) of treated Japanese children with influenza virus A (H3N2) infection and 16% (7/43) of treated Japanese children with influenza virus A (H1N1) infection, also with no evidence that these viruses transmitted efficiently (*12*,*13*).

In late January 2008, we reported an unexpected high level and unexpected spread of oseltamivir-resistant influenza viruses A (H1N1) (ORVs) in Europe caused by a H275Y (H274Y in N2 numbering) amino acid substitution in the neuraminidase (NA) of these viruses (*14*). Here, we analyze the distribution and transmission of ORVs in Europe during the winter of 2007–08, when influenza viruses A (H1N1) were the predominant circulating viruses in European countries (Table).

**Table:** Peak incidence rates of ILI or ARI infection for countries for which data were available, Europe, 2000–01 through 2007–08 influenza seasons*

Country	ILI/ ARI†	Peak incidence rate/10,000 population during influenza season							Peak incidence rates¶	
		2000–01 through 2007–08				Dominant virus§				
		Median	Range	Consecutive no. seasons‡					Incidence rate ratio	p value
						2000–01	2007–08			
Austria#	ILI	168.1	108.0–263.2	4		NA	186.1 (H1)			
Belgium	ILI	51.9	30.3–95.1	8		30.3 (H1)	38.0 (H1)		1.3	0.004
Bulgaria	ARI	NA				NA	186.0 (H1)			
Czech Republic	ARI	188.1	134.5–320.0	8		310.2 (H1)	144.4 (H1)		0.5	1.000
Denmark	ILI	30.7	13.8–47.8	8		44.5 (H1)	13.8 (H1)		0.3	1.000
Estonia	ILI	2.9	0.6–4.9	3		NA	2.9 (H1)			
France	ARI	336.1	279.7–448.8	7		NA	279.7 (H1)			
Germany	ARI	185.6	136.9–256.5	8		247.3 (H1)	136.9 (H1/B)		0.6	1.000
Greece	ILI	27.7	23.1–42.1	3		NA	23.1 (H1)			
Hungary	ILI	50.1	21.0–54.6	3		NA	54.6 (H1)			
Ireland	ILI	7.5	2.9–12.1	8		12.1 (H1)	4.9 (H1/B)		0.4	1.000
Italy	ILI	79.5	27.6–428.2	8		56.7 (H1)	72.1 (H1/B)		1.3	0.0001
Latvia	ILI	45.6	25.1–93.3	5		NA	26.6 (H1)			
Lithuania	ILI	34.9	13.3–47.2	7		NA	13.3 (H1)			
Luxembourg	ILI	72.6	32.7–79.1	5		NA	67.4 (H1)			
The Netherlands	ILI	10.3	6.6–24.0	8		6.9 (H1)	7.2 (H1/B)		1.0	0.400
Norway	ILI	18.5	10.9–31.7	3		NA	10.9 (H1/B)			
Poland	ILI	23.0	6.2–66.7	7		NA	16.6 (H1)			
Portugal	ILI	8.1	3.0–17.4	8		3.8 (H1)	6.2 (H1/B)		1.6	0.016
Romania	ILI	1.2	0.4–3.7	4		NA	1.4 (H1)			
Serbia	ILI	37.8	30.6–44.9	2		NA	30.6 (H1)			
Slovakia	ILI	136.3	49.5–337.3	8		337.3 (H1)	49.5 (H1)		0.1	1.000
Slovenia	ILI	15.2	4.5–39.2	8		14.1 (H1)	20.4 (H1)		1.5	0.001
Spain	ILI	21.2	4.2–54.1	8		4.2 (H1)	20.3 (H1/B)		4.8	0.0001
Sweden#	ILI	2.0	1.6–5.8	5		5.8 (H1)	1.8 (B)		0.3	1.000
Switzerland	ILI	39.8	19.4–53.2	7		NA	29.7 (H1)			
United Kingdom	ILI	3.8	2.7–8.4	8		5.5 (H1)	2.7 (H1/B)		0.5	1.000

*ILI, influenza-like illness; ARI, acute respiratory infection; NA, data not available. †For countries where both ILI and ARI data were available, only the ILI data are shown. ‡Leading up to 2007–08. §Dominant virus estimated on the basis of combined sentinel and nonsentinel data. The limits for codominant virus types/subtypes were 45%:55%. ¶2007–08 compared with 2000–01. The incidence rate ratio was calculated by dividing the peak incidence rate for 2007–08 by the peak incidence rate for 2000–01. If the p value estimated using z-statistics is <0.05, the incidence rate ratio is significantly >1, and therefore the peak incidence rate for 2007–08 is significantly higher than that for 2000–01. #Data for seasons 2002–03, 2003–04, and 2004–05 were missing.

## Methods

### Clinical Influenza Activity

The European Influenza Surveillance Scheme (EISS) actively monitored influenza activity from week 40 (October 1–7) of 2007 through week 19 (May 5–11) of 2008. EISS covers all 27 European Union countries plus Croatia, Norway, Serbia, Switzerland, Turkey, and Ukraine. In each country each week, 1 or several networks of sentinel general practitioners (GPs) reported rates of consultation for influenza-like illness (ILI) or acute respiratory infection (ARI) (*15*–*17*). ARI includes ILI and all other acute respiratory infections. For Croatia, Finland, Turkey, and Ukraine, no consultation data were available.

### Virologic Analysis

Sentinel GPs involved in clinical data recording of ILI or ARI also send nasal, pharyngeal, or nasopharyngeal specimens from a subset of their patients to the National Influenza Centers (NICs) for virus detection and characterization by using a variety of genetic or phenotypic methods (*18*–*20*). The NICs also analyzed specimens and influenza viruses obtained from other sources (e.g., from nonsentinel GPs, hospitals, or institutions). For Cyprus and Turkey, no virus detection data were available.

### Antiviral Drug Susceptibility Monitoring

Antiviral susceptibility data were generated either through the European Surveillance Network for Vigilance against Viral Resistance (VIRGIL) project at a single laboratory in London (UK Health Protection Agency) or directly by individual NICs by using methods described previously (*14*,*21*). Genetic analysis of virus isolates or clinical specimens was performed by using cycle-sequencing or pyrosequencing the NA gene, targeting the H275Y amino acid substitution in the N1 NA (*22*). The 50% inhibitory NAI concentration (IC_50_) of virus isolates was determined by using fluorescent or chemiluminescent enzyme assays (*23*,*24*). ORVs were defined as influenza viruses A (H1N1) with an IC_50_ >100 nmol/L for oseltamivir. Susceptibility to zanamivir was determined by using the same enzymatic method. Susceptibility to M2Is was determined by cycle-sequencing or pyrosequencing the M2 protein gene, targeting known resistance markers. Antiviral susceptibility data were not available for Cyprus, Lithuania, and Malta.

### Data Analysis

To obtain United Kingdom estimates, clinical and virologic surveillance data and antiviral susceptibility data were totaled for England, Northern Ireland, Scotland, and Wales. A single web-based European database at the EISS password-protected website (www.eiss.org) was used to collect antiviral susceptibility data and linked patient demographic and clinical data (*25*). Updates on possible resistant viruses were provided at regular intervals to EISS members, the World Health Organization, and the European Centre for Disease Prevention and Control.

The timing of the first week of continuous detection of influenza virus A and ORVs across Europe, both based on date of specimen collection, were analyzed by linear regression analysis using center longitude and center latitude of a country as explanatory variables. A maximum interruption of 1 week with no influenza virus A or ORV detection was allowed in estimating the first week of continuous detection. The average European delay between the first week of continuous detection of influenza virus A and of ORV was calculated as the average of the differences in number of weeks between both, by country.

The analysis of temporal trends in the prevalence of ORVs in countries and for Europe was confounded by different levels of sampling in different countries (*18*), enhanced antiviral susceptibility testing in some countries, and lack of data on the proportion of ORVs for some or most weeks for several other countries. To ensure a more representative picture of temporal trends in the proportion of ORVs, a mixed effect logistic regression modeling approach (*26*,*27*) was used, which allows modeling of binomial proportions, i.e., a numerator and a denominator as a function of time, where the coefficients of this function are allowed to vary for each country around a mean value, combining data from all countries. If there are no observations or the denominator is small, the fit will shrink to its overall mean, and uncertainties increase. Three fractions were modeled: “ILI per population covered,” “influenza A virus detections per specimens tested,” and “A (H1N1) resistant per A (H1N1) tested.” By multiplying the first 2 fractions by the total population, we obtained the number of patients with ILI who had influenza A in a country. By dividing this number by the sum of the number of patients with ILI who had influenza A for all countries, we obtained the relative weights. By multiplying the weights with the prevalences of ORVs summed over all countries, we obtained the weekly European prevalences of ORVs. The modeled weekly prevalences of ORVs were subsequently used to calculate the average prevalence of ORVs by country and for Europe (Technical Appendix).

We performed all statistical analyses by using the software package R version 2.8.0 (*28*). Box-and-whisker plot analysis was used to select viruses with outlying high IC_50_ values for further analysis (*7*,*29*). For oseltamivir outlier identification, all viruses defined as resistant for oseltamivir (IC_50_ >100 nmol/L) were first removed. Minor outliers were defined as values lying between the upper quartile (UQ) + 1.5 × interquartile region (IQR) and UQ + 3 × IQR; major outliers were defined as values lying above UQ + 3 × IQR, based on analysis of all viruses in a particular subtype over a particular winter season.

Phylogenetic analysis of NA and hemagglutinin (HA) gene sequences used maximum parsimony (PAUP* version 4.0; Sinauer Associates, Sunderland, MA, USA). Sequences of ORVs and oseltamivir-sensitive influenza A (H1N1) viruses (OSVs) were chosen as representative of influenza viruses A (H1N1) isolated during the 2007–08 influenza season (i.e., weeks 40–52 of 2007 and weeks 1–19 of 2008) in different European countries and a few from other regions of the world and were compared with those of a few influenza viruses A (H1N1) isolated before the 2007–08 season, including sporadically isolated ORVs. GenBank accession numbers are listed in the  Appendix Table.

## Results

### Seasonal Surveillance

**Figure 2:**
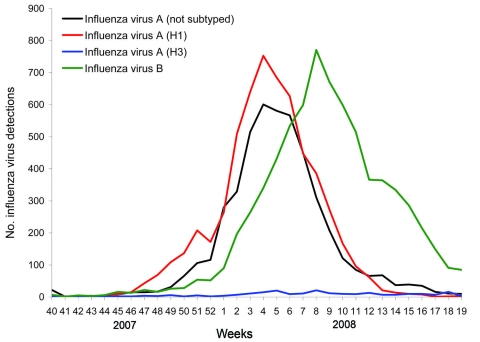
Total number of influenza virus detections, by type and subtype and by week, Europe, winter 2007–08.

The 2007–08 influenza season in Europe was initially dominated by influenza viruses A (n = 10,720; 60% of all influenza virus detections). Influenza viruses B (n = 7,150; 40% of all influenza virus detections) became dominant in week 8 (Figure 2). Of the 5,984 (56%) influenza viruses A subtyped, 5,748 (96%) were H1, and 236 (4%) were H3. Overall, influenza virus detections peaked in week 6, in week 4 for influenza viruses A (H1N1), and in week 8 for influenza viruses B. Of the 2,136 influenza viruses A (H1N1) characterized antigenically, 97% were reported to be closely related to the vaccine strain A/Solomon Islands/3/2006, although half of these viruses were reported to be more closely related to A/Brisbane/59/2007, the vaccine strain recommended for the 2008–09 season (*30*).

The first countries in Europe where influenza viruses A started to circulate continuously were France, Spain, Switzerland, and the United Kingdom in week 40. Spatial analysis of the timing of the first week of continuous detection of influenza viruses A across Europe (n = 30 countries) showed a west-to-east pattern: estimated parameter for longitude was 0.261 weeks per degree longitude (95% confidence interval [CI] 0.138–0.385, p = 0.001), and for latitude –0.108 weeks per degree latitude (95% CI –0.324 through 0.108, p = 0.366), with R^2^ = 0.32 for the linear regression fit.

### Antiviral Drug Susceptibility

**Figure 3:**
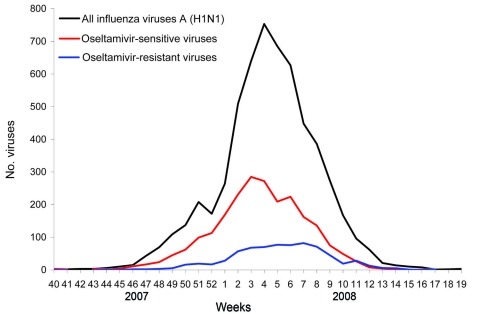
Total influenza A viruses subtyped as H1N1 and number of oseltamivir-resistant or oseltamivir-sensitive viruses among the subset of influenza viruses A (H1N1) for which oseltamivir susceptibility was determined, by week, Europe, winter 2007–08.

The estimated number of influenza viruses A (H1N1) among all detected influenza viruses A (n = 10,720) was 10,291 following extrapolation from the proportion of 96% influenza viruses A (H1N1) among all 5,984 subtyped influenza viruses A. Of the 10,291 influenza viruses A (H1N1), 2,949 (29%) were tested for antiviral susceptibility, 1,080 by both phenotypic assay (IC_50_) and sequencing, 601 by phenotypic assay alone, and 1,268 by sequencing alone. Of the 2,949 viruses tested, 712 (24%) were oseltamivir resistant either by presence of the H275Y substitution (n = 548) or an IC_50_ >100 nmol/L for oseltamivir (n = 463) (Figure 3). Correlation was 100% between sensitive phenotype (IC_50_ <100 nmol/L) and the presence of H275 (n = 781) and between resistant phenotype (IC_50_ >100 nmol/L) and the presence of Y275 (n = 299). OSVs (n = 1,218) had a median IC_50_ of 1.7 nmol/L for oseltamivir (range 0.1 nmol/L–23.2 nmol/L) and only 9 minor outliers (thresholds IC_50_ >12.0 nmol/L and <53.1 nmol/L) were identified. ORVs (n = 463) had a median IC_50_ of 653 nmol/L (range 140 nmol/L–4,000 nmol/L). None of the 429 phenotypically characterized ORVs showed evidence of resistance to zanamivir (median IC_50_ 1.8 nmol/L, range 0.2 nmol/L–25.8 nmol/L), and only 17 minor outliers (thresholds IC_50_ >8.5 nmol/L and <27.5 nmol/L) were identified. None of 237 ORVs tested for M2I sensitivity had any of the common resistance substitutions in the M2 protein.

**Figure 4:**
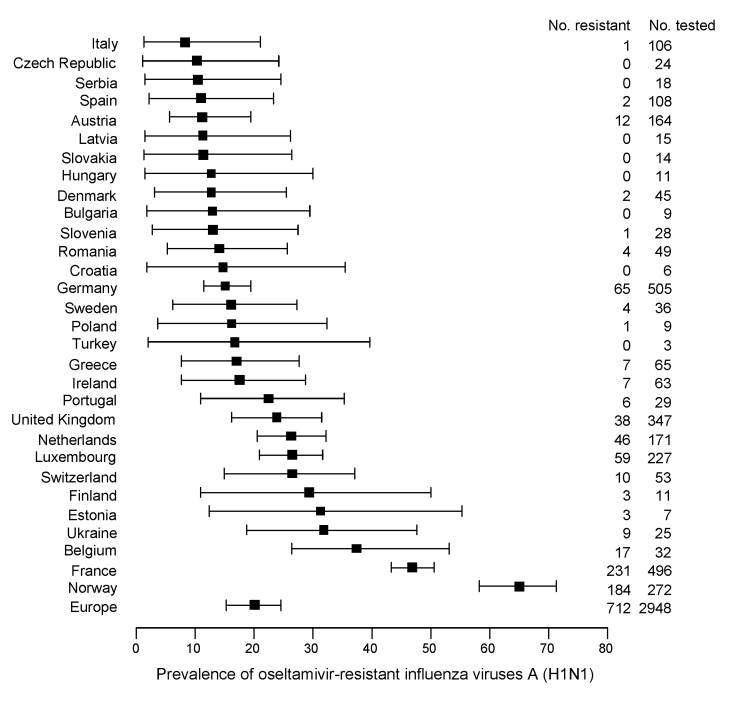
Modeled average prevalence of oseltamivir-resistant influenza viruses A (H1N1), with 95% confidence intervals (error bars), ranked by country, Europe, winter 2007–08. Text columns on the right list the absolute cumulative number of oseltamivir-resistant influenza viruses A (H1N1) and number of influenza viruses A (H1N1) tested for oseltamivir susceptibility per country.

ORVs were detected in 22 of the 30 countries for which susceptibility data were available, with Norway having the highest proportion of ORVs (Figure 4). Modeling showed the overall average prevalence of ORVs by country ranged from 8.3% (95% CI 1.3%–21%) in Italy to 65.0% (95% CI 58.2%–71.3%) in Norway; for Europe, the average prevalence of ORVs was 20.1% (95% CI 15.2%–24.6%).

The earliest detection of ORVs was in France and the United Kingdom in week 46 and in Norway in week 47. Countries where continuous detection of ORVs first began included Norway in week 47, France in week 49, the United Kingdom in week 51, and the Netherlands in week 52. Spatial analysis of the timing of the first week of continuous ORV detection across Europe (n = 14 countries) showed a west-to-east trend pattern: estimated parameter for longitude was 0.156 weeks per degree longitude (95% CI 0.033–0.280, p = 0.031), and for latitude 0.007 weeks per degree latitude (95% CI –0.209 through 0.223, p = 0.953), with R^2^ = 0.36 for the linear regression fit. The average delay between the first week of continuous detection of influenza virus A and continuous detection of ORV was 5.7 weeks (range 0–15, 95% CI 2.8–8.4).

**Figure 5:**
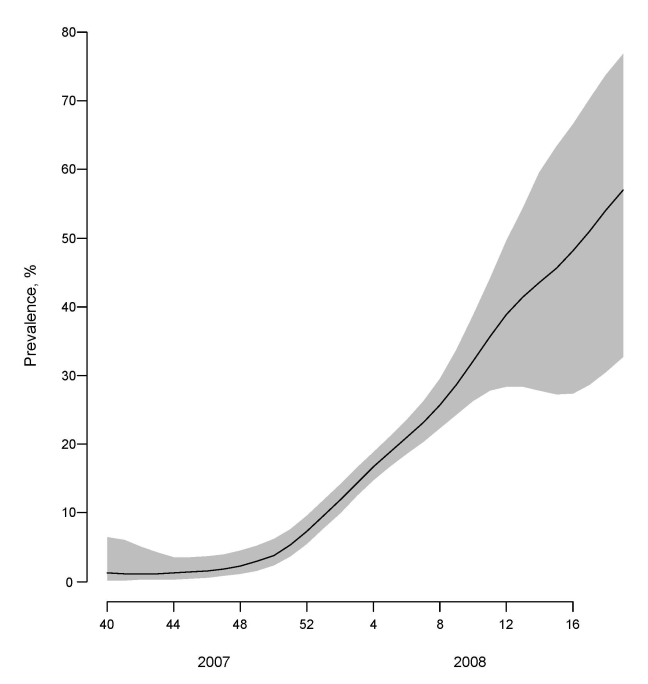
Weighted average prevalence of oseltamvir-resistant influenza viruses A (H1N1), Europe, winter 2007–08. The light gray region indicates the 95% confidence interval.

Modeling showed a gradual increase for Europe in prevalence of ORVs over time, from close to 0 in week 40 to ≈56% in week 19 (Figure 5). This overall increase reflected prevalence increases in most individual countries in addition to Norway where the modeled prevalence started high at ≈60% and remained so throughout the period of virus circulation (Appendix Figure. Outside the main influenza virus A (H1N1) outbreak period, from week 51 to week 10 (Figure 2), the CIs for the prevalence of ORVs by country and for Europe were wide (Figure 5; Appendix Figure) because of the low numbers of influenza virus A (H1N1) detected or analyzed for antiviral resistance (Technical Appendix).

### Phylogenetic Analysis

**Figure 6:**
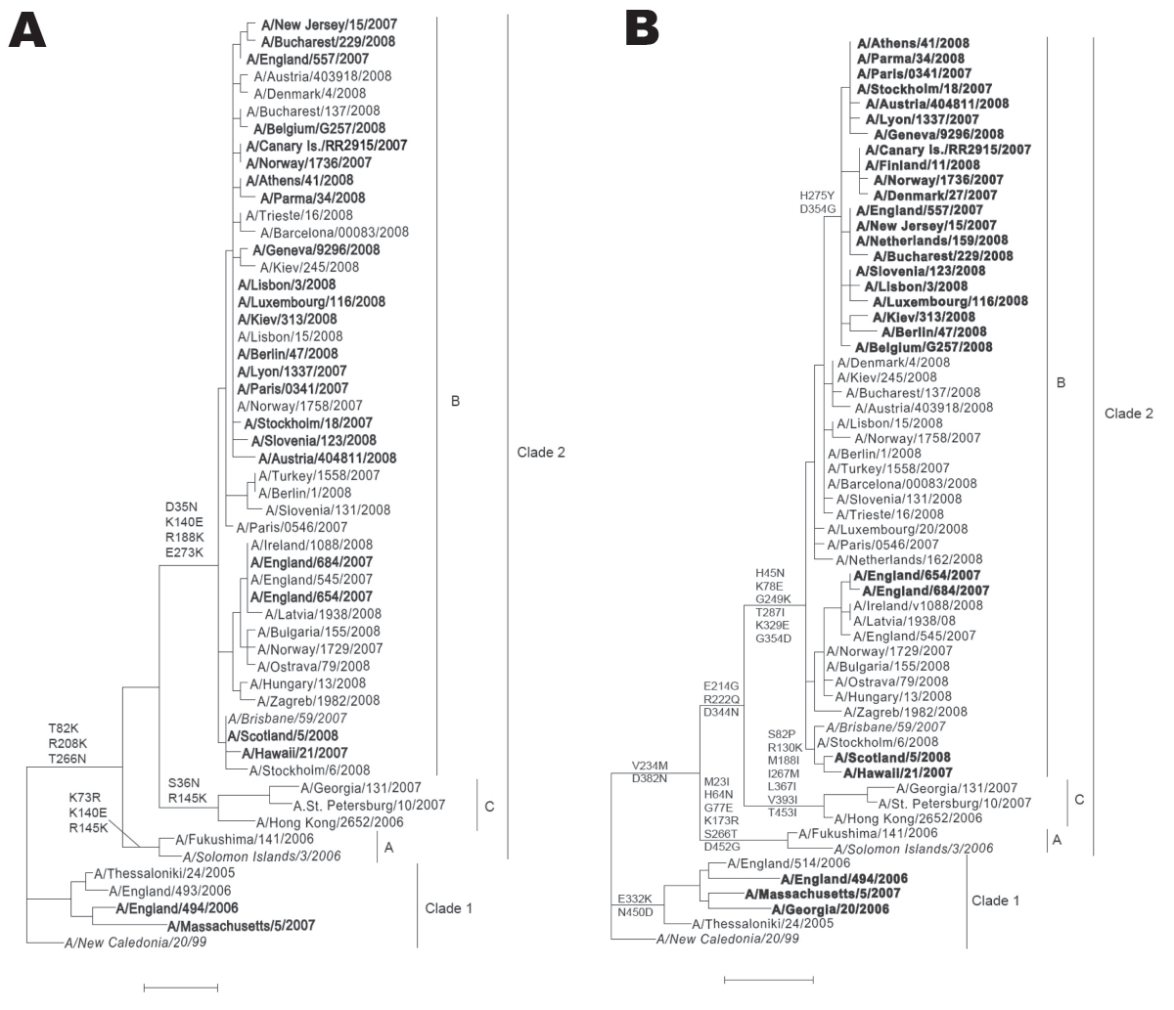
Phylogenetic comparisons of the hemagglutinin (A) and neuraminidase (B) genes of influenza viruses A (H1N1). Sequences of oseltamivir-resistant viruses, possessing the H275Y (H274Y in N2 numbering) mutation are in **boldface**; vaccine strains are in *italics*. Common amino acid changes that distinguish clades 1 and 2 and subgroups of clade 2 are shown. Scale bars indicate 0.01 nucleotide substitutions per site.

Phylogenetic comparisons of HA and NA genes showed that the sequences of most recent European influenza viruses A (H1N1) fell within clade 2B, represented by A/Brisbane/59/2007, the recently recommended vaccine virus for 2008–09 (Figure 6). The NA sequences of most European ORVs form a cluster, characterized by a difference in amino acid residue 354 (D354G), as well as 275 (H275Y) compared with OSVs, including some ORVs from the United States and Japan (*30*,*31*). A degree of heterogeneity was observed, especially among ORVs from the United Kingdom; however, the NA sequences in these smaller clusters, represented by, for example, A/Scotland/5/2008 (and A/Hawaii/21/2007) or A/England/654/2007, are not distinguished from those of OSVs by any common amino acid differences other than H275Y. Some of these sequences fall close to those of ORVs recently isolated in Japan (*31*). The corresponding HA gene sequences within clade 2B, however, did not exhibit segregation complementary to that for NA gene sequences and no common amino acid changes distinguished ORVs and OSVs (Figure 6). Although the D344N substitution in NA has been associated with increases in the enzyme activity (*32*), this amino acid is common to both clades 2B and 2C, and none of the clade-specific differences between the NA (13 amino acids) or HA (6 amino acids) can readily account for the greater proportion of ORVs in clade 2B over clade 2C viruses.

## Discussion

Unexpectedly, influenza viruses A (H1N1) with a single amino acid substitution H275Y in the NA, which caused a several hundred-fold selective reduction in susceptibility to oseltamivir, emerged and were sustained in circulation in Europe during 2007–08, despite low antivirual drug use (Figure 1). Before the 2007–08 season, <1% of viruses tested since the start of European antiviral surveillance in 2004 had IC_50_ values >100 nmol/L for NAI drugs (A. Lackenby et al., unpub. data), in concordance with results from worldwide surveillance (*8*,*9*). In 2007–08, influenza viruses A (H3N2) and B circulating in Europe remained sensitive to NAI drugs.

This emergence of oseltamivir-resistant influenza virus A (H1N1) in Europe coincided with the dominant circulation of this virus subtype during the 2007–08 winter in Europe and the emergence of a new drift variant, A/Brisbane/59/2007 (*30*). Of the last 12 influenza seasons, influenza viruses A (H1N1) were dominant only in 2000–01, which included a new drift variant, A/New Caledonia/20/99 (*20*). In the other 10 seasons, influenza viruses A (H1N1) played a minor role, with influenza viruses A (H3N2) dominant in 9 seasons. Compared with 2000–01, peak incidence rates for ILI or ARI in 7 of 13 countries were similar or lower in 2007–08 (Table). In 6 countries, the peak incidence rates were significantly higher in 2007–08 than in 2000–01, but with a <2-fold difference in 5 countries and, in Spain only, a 4.8-fold difference. Both the 2000–01 and 2007–08 seasons were unremarkable in the overall clinical impact of influenza, with normal seasonal activity as measured by comparison of peak incidence rates for all seasons since 2000–01.

Sporadically occurring A/New Caledonia/20/99-like ORVs with H275Y were detected during the 2006–07 season in the United Kingdom and United States but did not become epidemiologically important. Indeed, the genetic background plays a role in retaining the replication efficiency and pathogenicity of recombinant influenza viruses A (H5N1) and A (H1N1) after introduction of tyrosine at position 275 (*33*). Furthermore, other previously analyzed influenza viruses A (H1N1) with the H275Y mutation showed impaired replicative ability in cell culture and reduced infectivity and substantially compromised pathogenicity in animal models, compared with the corresponding wild-type virus (*34*,*35*). The coincidental emergence of H275Y with the circulation of the A/Brisbane/59/2007 drift variant may have favored the emergence of fit transmissible ORVs. This point is also illustrated by the emergence of A/Brisbane/59/2007-like ORVs in other parts of the Northern Hemisphere and their continued circulation during the 2008 Southern Hemisphere influenza epidemic season (*36*–*38*). Since the last quarter of 2007, ORVs have been detected in continents other than Europe, with proportions of ORVs varying from 100% in South Africa and Australia to <5% in Japan. Trend data are limited: a slight monthly increase was noted in China/Hong Kong and Japan; in Canada, the increase was similar to that in Europe, from 0% ORVs in November 2007 to 86% ORVs in April 2008 (*36*).

Using modeling, we showed that the prevalence of ORVs increased in the European region from ≈0% at the start to 56% at the end of the season. The finding of a high prevalence of ORVs in the community and the overall temporal increase in resistance demonstrates that the previously documented reduced fitness of viruses bearing the H275Y mutation, ostensibly caused by structural and functional constraints (*10*), has been overcome in currently circulating influenza viruses A (H1N1). The results of Rameix-Welti et al. (*32*) suggest that a combination of specific amino acid substitutions have increased the affinity of the NA of recent influenza viruses A (H1N1) (ORVs and OSVs) for substrate. A better balance of NA and HA activities in ORVs compared with OSVs may have contributed to the overall fitness and transmissibility of ORVs. However, growth curves conducted in tissue culture of pairs of ORVs and OSVs demonstrated no differences in growth kinetics or final virus yields. Therefore, changes in other genes also may be involved in the overall impact on the fitness of ORVs, for which whole genome sequencing is necessary.

For Europe, no focal point of initiation of spread could be identified. The spread of ORV from west to east paralleled that of influenza virus A in Europe, and there was an average delay of 5.7 weeks for the appearance of ORVs after the start of influenza virus A circulation. However, the low R^2^ values for both patterns make definitive conclusions difficult to draw about the spatial spread of either influenza viruses A or ORVs. Several independent introductions into European countries of a sensitive and a resistant strain might explain the low R^2^ values.

Estimating whether a global focal point exists from which ORVs emerged to spread to the rest of the world is not possible, but the fact that Japan, the country with the highest per capita use of oseltamivir (*5*), had relatively low levels of circulating ORVs during the 2007–08 influenza season is relevant and reflects the limited circulation of the clade 2B A/Brisbane/59/2007-like viruses belonging to the European cluster in this region (*31*,*36*).

The close relationships between the NA sequences of most of the 2007–08 European ORVs and their segregation from those of OSVs suggest that resistance results in large part from the spread of a single variant. Phylogenetic analyses show that this is a property of clade 2B A/Brisbane/59/2007-like viruses and is not associated with emergence of another antigenic variant. However, identification of other resistant variants in the United Kingdom, some of which are more closely related to OSVs than to most ORVs (e.g., A/England/654/2007) indicates the independent parallel emergence of multiple resistant variants. This is emphasized by small distinct clusters of closely related ORVs in Japan that are related to European OSVs, whereas only a few of the Japanese ORVs belonged to the large European ORVs cluster (*31*). Resolution of the origin and frequency of emergence of ORVs and association with drug use clearly require substantially more intimate knowledge of the genetic relationships among OSVs and ORVs worldwide. Our observations suggest that the new genetic background of influenza viruses A (H1N1) that appeared in 2007 enabled the virus to develop oseltamivir resistance independently at several locations in the world.

The combined effect of the relatively high level of circulation of influenza viruses A (H1N1) in Europe; the introduction of a new antigenic drift variant in a susceptible population, partly related to the lack of substantial influenza virus A (H1N1) circulation since the 2000–01 season; and the uncompromised transmissibility of the ORVs contributed to the epidemiologic success of the ORVs during the 2007–08 season. This phenomenon shows clearly that continuation of antiviral susceptibility monitoring and increasing capacity for timely response are essential (*21*,*39*). In addition, the appearance of viable transmitting ORVs is a reminder that the level of resistance to oseltamivir of seasonal or pandemic virus cannot be predicted, and therefore antiviral strategies should not rely on single drugs (*40*). Although oseltamivir remains a valuable influenza antiviral agent, the emergence of natural resistance shifts attention from oseltamivir to other antiviral agents and to improved vaccination (e.g., greater vaccination coverage, more immunogenic and broadly reacting vaccines) in the fight against seasonal and pandemic influenza.

## Supplementary Material

Appendix Figure Fitted curves to the proportion oseltamivir-resistant viruses among influenza viruses A (H1N1) tested for resistance (both sentinel and nonsentinel) for all countries for which data were available for inclusion in modeling the European trend (see Figures 4 and 5). The x axes display the week in which the clinical specimens were collected (weeks 40-52 of 2007 and weeks 1-19 of 2008). The y axes display the percentage oseltamivir resistant influenza viruses A (H1N1). Plus signs indicate the actual determined proportions resistant A (H1N1) viruses; light gray region is the 95% confidence interval of the model.

Appendix Table GenBank accession numbers of hemagglutinin and neuraminidase sequences used in the phylogenetic Analyses.

Technical Appendix Statistical Analysis of Temporal Trends of Resistant Influenza A (H1N1) Viruses, Europe.
