# Supplementary figures and images for: Oseltamivir-Resistant Influenza Virus A (H1N1), Europe, 2007–08 Season
Source: Emerg Infect Dis. 2009 Apr;15(4):552–60. doi: 10.3201/eid1504.081280 (PMC2671453; doi:10.3201/eid1504.081280)

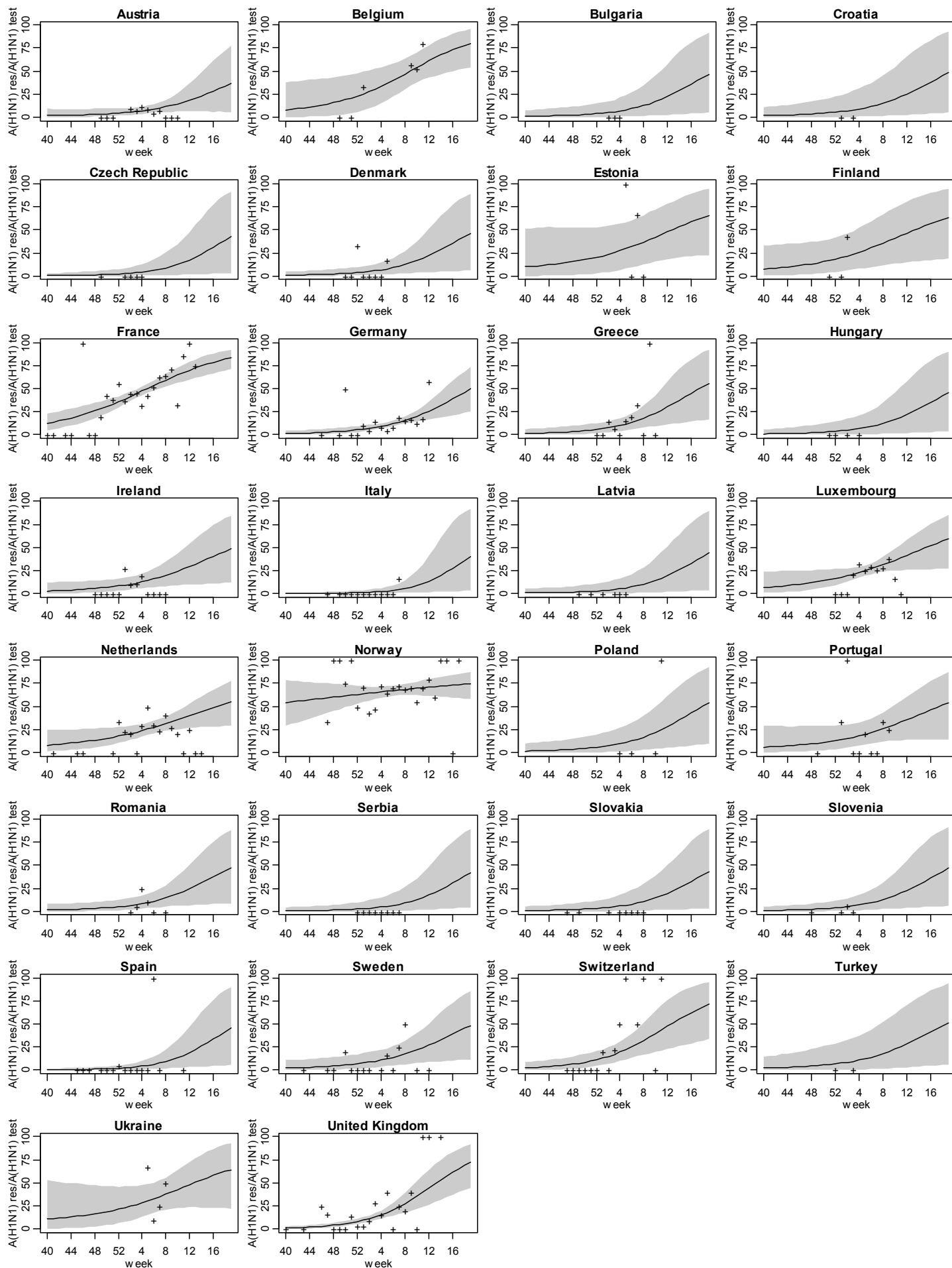

Supplement: Appendix Figure — Fitted curves to the proportion oseltamivir-resistant viruses among influenza viruses A (H1N1) tested for resistance (both sentinel and nonsentinel) for all countries for which data were available for inclusion in modeling the European trend (see Figures 4 and 5). The x axes display the week in which the clinical specimens were collected (weeks 40-52 of 2007 and weeks 1-19 of 2008). The y axes display the percentage oseltamivir resistant influenza viruses A (H1N1). Plus signs indicate the actual determined proportions resistant A (H1N1) viruses; light gray region is the 95% confidence interval of the model. [file 08-1280_appF-s1.pdf]
