# Supplementary material for: Oseltamivir-Resistant Influenza Virus A (H1N1), Europe, 2007–08 Season
Source: Emerg Infect Dis. 2009 Apr;15(4):552–60. doi: 10.3201/eid1504.081280 (PMC2671453; doi:10.3201/eid1504.081280)
Supplement: Appendix Table — GenBank accession numbers of hemagglutinin and neuraminidase sequences used in the phylogenetic Analyses. [file 08-1280_appT-s2.pdf]

Appendix Table. GenBank accession numbers of hemagglutinin and neuraminidase sequences used in the phylogenetic analyses

Strain	Hemagglutinin*	Neuraminidase
A/Athens/41/2008	FJ654307	FJ403554
A/Austria/403918/2008	FJ654334	FJ403581
A/Austria/404811/2008	FJ654331	FJ654335
A/Barcelona/00083/2008	FJ654312	FJ403560
A/Belgium/G257/2008	FJ654300	FJ403562
A/Berlin/1/2008	FJ654311	FJ403557
A/Berlin/47/2008	FJ654333	FJ403568
A/Brisbane/59/2007	CY030230	CY030233
A/Bucharest/137/2008	FJ654316	FJ403576
A/Bucharest/229/2008	FJ654323	FJ403548
A/Bulgaria/155/2008	FJ654313	FJ403566
A/Canary Islands/RR2915/2007	FJ654319	FJ403570
A/Denmark/4/2008	FJ654337	FJ403588
A/Denmark/27/2007	ND	FJ403583
A/England/493/2006	FJ445027	Not included
A/England/494/2006	FJ445085	FJ445080
A/England/514/2006	Not included	FJ445081
A/England/545/2007	FJ445044	FJ445087
A/England/557/2007	FJ445089	EU624316
A/England/654/2007	FJ445090	FJ445025
A/England/684/2007	FJ654338	FJ403586
A/Finland/11/2008	ND	FJ380948
A/Fukushima/141/2006	FJ654301	FJ403584
A/Geneva/9296/2008	FJ654332	FJ403574
A/Georgia/20/2006	ND	EU516197
A/Georgia/131/2007	FJ654303	FJ403553
A/Hawaii/21/2007	EU516080	EU516112
A/Hong Kong/2652/2006	CY031342	FJ403585
A/Hungary/13/2008	FJ654320	FJ403564
A/Ireland/v1088/2008	FJ654327	FJ403572
A/Kiev/245/2008	FJ654330	FJ403578
A/Kiev/313/2008	FJ654315	FJ403561
A/Latvia/938/2008	FJ654329	FJ403569
A/Lisbon/3/2008	FJ654310	FJ403558
A/Lisbon/15/2008	FJ654318	FJ403579
A/Luxembourg/20/2008	ND	FJ403565
A/Luxembourg/116/2008	FJ654328	FJ403582
A/Lyon/1337/2007	CY031384	FJ403553
A/Massachusetts/5/2007	EU516110	EU516028
A/Netherlands/159/2008	ND	FJ445020
A/Netherlands/162/2008	ND	FJ445021
A/New Caledonia/20/99	CY031336	AJ518023
A/New Jersey/15/2007	EU516083	EU885517
A/Norway/1729/2007	FJ654306	FJ403552
A/Norway/1736/2007	FJ654304	FJ403550
A/Norway/1758/2007	FJ654305	FJ403551
A/Ostrava/79/2008	FJ654314	FJ403559
A/Paris/0341/2007	EU551832	EU551811
A/Paris/0546/2007	EU551837	EU551822
A/Parma/34/2008	FJ654324	FJ403575
A/Scotland/5/2008	FJ654326	FJ403587
A/Slovenia/123/2008	FJ654322	FJ403563
A/Slovenia/131/2008	FJ654321	FJ403571
A/Solomon Islands/3/2006	EU100724	EU124136
A/Stockholm/6/2008	FJ654339	FJ403546
A/Stockholm/18/2007	FJ654308	FJ403547
A/St. Petersburg/10/2007	FJ654302	FJ403549
A/Thessaloniki/24/2005	FJ654336	FJ403567
A/Trieste/16/2008	FJ654309	FJ403556
A/Turkey/1558/2007	FJ654317	FJ403577
A/Zagreb/1982/2008	FJ654325	FJ403573

* ND, not determined.
