# Supplementary material for: Oseltamivir-Resistant Influenza Virus A (H1N1), Europe, 2007–08 Season
Source: Emerg Infect Dis. 2009 Apr;15(4):552–60. doi: 10.3201/eid1504.081280 (PMC2671453; doi:10.3201/eid1504.081280)
Supplement: Technical Appendix — Statistical Analysis of Temporal Trends of Resistant Influenza A (H1N1) Viruses, Europe. [file 08-1280_Techapp-s3.pdf]

Oseltamivir-Resistant Influenza Virus A (H1N1), Europe, 2007–08 Season

Technical Appendix

Statistical Analysis of Temporal Trends of Resistant Influenza A (H1N1) Viruses, Europe

The goal of the statistical analysis is to estimate a European weighted average prevalence of oseltamivir-resistant influenza A viruses (H1N1) among all detected influenza viruses A (H1N1) for the 2007–08 influenza season (week 40 of 2007 through week 19 of 2008). This prevalence can be calculated for each country and for each week. By using weighting techniques, a European prevalence can be obtained.

Datasets Used

The first dataset used was weekly sentinel surveillance data for influenza-like illness (ILI) for most countries or for acute respiratory infections (ARI) for Bulgaria, France, and Germany, based on the week of consultation of the sentinel physician by the patient. The ARI data were corrected to ILI data by methods described below. The number of clinical cases, the number of patients in the patient lists of the sentinel physicians, and the total population data per country for 2008 (obtained from the Statistical Office of the European Communities; http://epp.eurostat.cec.eu.int) were included in the analysis.

The second dataset used was weekly sentinel surveillance for viral diagnosis data (type and subtype of virus) of patients with ILI, based on the week the clinical specimens were taken. Nonsentinel virus detections were not taken into account because no denominator data were available for these virus detections. In addition, sentinel virus detections are derived from the same population from which the clinical incidence data were derived. Virus detection data derived from patients with ARI in Czech Republic, Bulgaria, and France were corrected to ILI data by methods described below. Because not all countries reported subtyping data of influenza A viruses for the full dataset and the overall European prevalence of A (H1N1) viruses among

influenza A viruses was estimated at 96%, we assumed that for any given week and in any country all influenza virus A detections were A (H1N1). Therefore, the number of specimens analyzed and the number of specimens with influenza virus A were included in the analysis.

The third dataset used was weekly susceptibility data of influenza viruses A (H1N1) for oseltamivir derived from sentinel and nonsentinel (e.g., from hospital or peripheral laboratories) sources combined, based on the week the clinical specimens were taken. The number of influenza viruses A (H1N1) analyzed and the number of oseltamivir-resistant influenza viruses A (H1N1) were included in the analysis. Because the European crude proportion of resistant influenza viruses A (H1N1) among the tested influenza viruses A (H1N1) was similar between viruses from sentinel and nonsentinel sources, no distinction was made between sentinel and nonsentinel viruses to estimate the fraction of patients with resistant influenza virus A (H1N1) per number of patients with A virus (H1N1) tested for resistance.

A fourth dataset was used to model the correction of viral diagnosis data based on specimens collected from ARI patients to diagnosis data on the basis of specimens collected from patients with ILI. This fourth dataset was collected in the Dutch sentinel surveillance during 3 subsequent seasons (ARI-EL study), 2000–01, 2001–02, and 2002–03, and consisted of consultation rates for ILI and acute respiratory tract infections other than ILI (ARTI) and virus detections in specimens collected from patients with ILI or ARTI (*1*).

Countries were excluded from the modeling if $\geq$1 of the first 3 datasets described above were missing. In the first dataset, these were Croatia, Cyprus, Finland, Malta, Turkey, and Ukraine; in the second, Cyprus and Turkey; and in the third, Cyprus, Lithuania, and Malta.

Methods and Results

Calculation of an average prevalence would have been relatively simple if for each week all datasets were complete for each country. However, only limited data are available. Especially at the beginning and end of the season, data are missing. These missing data occur, for example, in the number of patients with ILI or ARI, in the number of specimens tested for influenza virus detection and subtype determination, and especially in the number of influenza viruses A (H1N1) tested for oseltamivir resistance. To correct for these missing data, a modeling approach was used.

For the weighting procedure, a target population must be identified first. For example, it is not correct to weight directly with the number of inhabitants of a country because not every person is infected. The target population is identified as follows. A fraction of the total population of a country is covered by the sentinel physicians. This population is divided into "ILI" and "non-ILI" groups. The ILI population is the first part of the target population. From a fraction of the ILI sentinel population, specimens are tested for influenza virus. This population is divided into "influenza A" and "non-influenza A" groups. The influenza A population is the second part of the target population, so the target population is ILI patients who are infected with influenza A virus. Within this target population, from a limited number of persons, the virus is tested for resistance; this fraction varies largely by country and week. We assume that sentinel data are representative of the whole population in a country, that all influenza viruses A are A (H1N1), and that the prevalence of resistance among tested A viruses (H1N1) does not depend on testing of sentinel or nonsentinel specimens. Hence, a total of 3 fractions have to be modeled: "ILI per population covered," "influenza A sentinel per specimens sentinel," and "A (H1N1) resistant per A (H1N1) tested."

The used model is a so-called mixed effect logistic regression model (*2,3*). This model allows modeling of binomial proportions, i.e., a numerator and a denominator as a function of time:

$$y_{i,j} \sim Bin(n_{i,j}, p_{i,j})$$

$$\log\left(\frac{p_{i,j}}{1-p_{i,j}}\right) = b_{0,i} + b_{1,i}t_j + b_{2,i}t_j^2 + b_{3,i}t_j^3$$

For country i at time j the number of cases $y_{i,j}$ comes from a binomial distribution with parameters $n_{i,j}$, the denominator, and $p_{i,j}$, the proportion. The log-odds are parametric functions of time, where the parameters $b_{0,i}$, …, $b_{3,i}$ themselves come from a multivariate normal distribution (here, 4 parameters). As a result, each country has its own parameters that vary around a mean value β_0, …, β_3:

$$\begin{pmatrix} b_{0,i} \\ b_{1,i} \\ \vdots \\ b_{3,i} \end{pmatrix} \sim MVN\left[\begin{pmatrix} \beta_0 \\ \beta_1 \\ \vdots \\ \beta_3 \end{pmatrix}, \begin{pmatrix} \sigma_{0,0}^2 & \sigma_{0,1}^2 & \cdots & \sigma_{0,3}^2 \\ \sigma_{1,0}^2 & \sigma_{1,1}^2 & \cdots & \sigma_{1,3}^2 \\ \vdots & \vdots & \ddots & \vdots \\ \sigma_{3,0}^2 & \sigma_{3,1}^2 & \cdots & \sigma_{3,3}^2 \end{pmatrix}\right]$$

The advantage of such an approach is that it smartly combines data from all countries. The larger the denominator, the more information an observation provides to the estimation of *b*. As a consequence, if there are no observations, or the denominator is small, the fit will shrink to its mean value *β*, and uncertainties increase. Using this modeling approach, estimating the weekly prevalences still is possible, even if there are no observations.

For the countries collecting ARI clinical data, the fractions "ARI per population covered" were pragmatically converted to "ILI per population covered" by multiplying the results by a modeled weekly fraction ILI/ARI for a "mean" country on the basis of data from countries with both weekly clinical ILI and ARI sentinel surveillance data (Belgium, Czech Republic, Estonia, Latvia, Lithuania, Luxembourg, Romania, Slovakia, and Slovenia). The fraction "influenza A sentinel/specimens sentinel" for the countries collecting virus detection data from patients with ARI were pragmatically converted to ILI by multiplying the results by a modeled weekly factor for a mean influenza season, based on the Dutch ARI-EL dataset (*1*).

The results of the individual steps taken to estimate a European weighted average prevalence are shown in Figures 1–5 (if available for a country, otherwise the country is not shown). Averaged over the whole season, the European prevalence is 20.1% (95% CI, 15.2%–24.6%).

References

1. van Gageldonk-Lafeber AB, Heijnen ML, Bartelds AI, Peters MF, van der Plas SM, Wilbrink B. A case-control study of acute respiratory tract infection in general practice patients in the Netherlands. Clin Infect Dis. 2005;41:490–7.
2. Molenberghs G, Verbeke G. 2006. Models for discrete longitudinal data, 2nd ed. Series: Springer Series in Statistics, Springer.

3. Gelman A. Hill J. 2007. Data analysis using regression and multilevel/hierarchical models. Cambridge University Press.

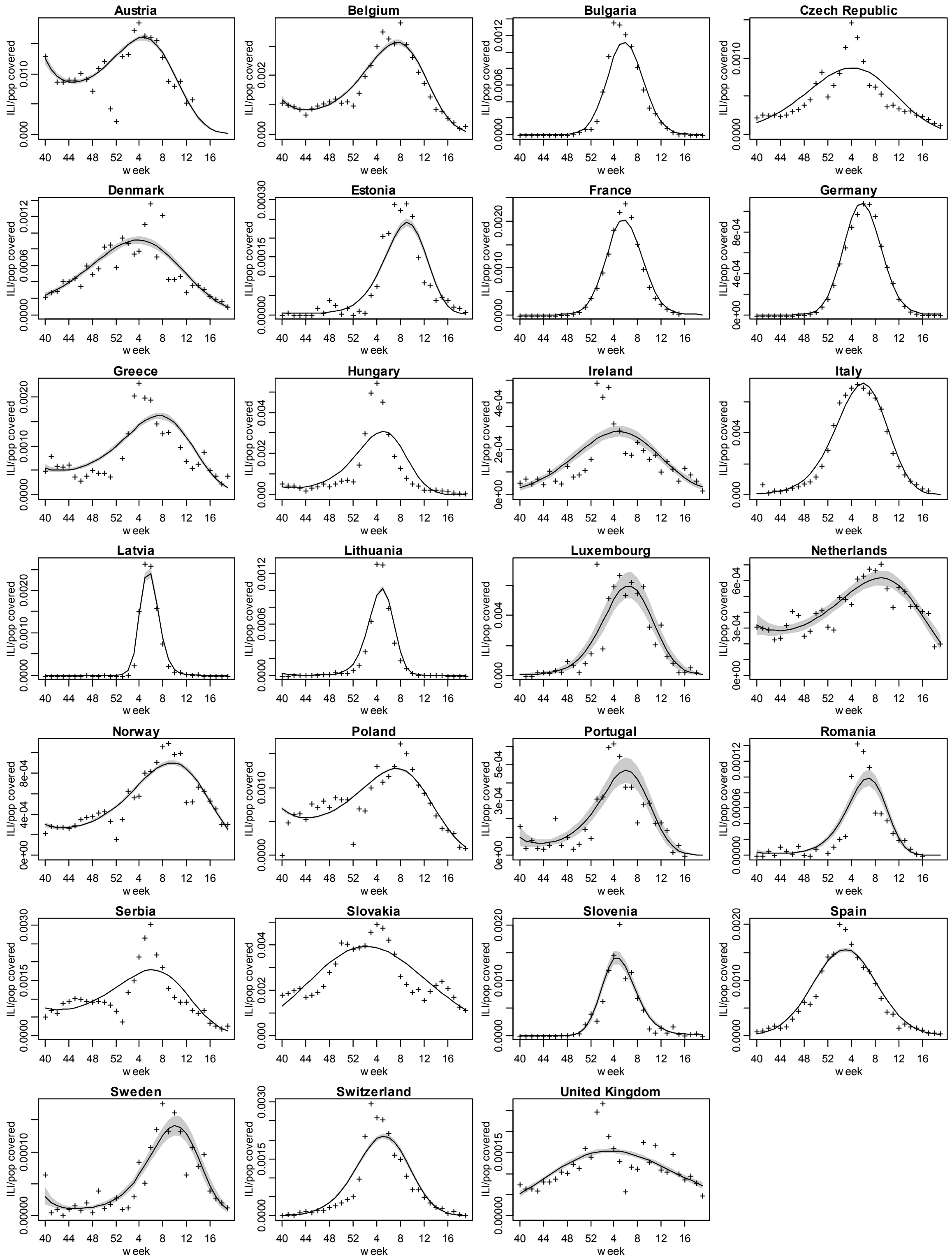

Technical Appendix Figure 1. Fitted curves to the percentage of influenza-like illness (ILI) per population covered by sentinel physicians. Light gray area is the 95% confidence interval, which is small because of the large denominators. If these fractions are multiplied by the total population of a specific country, the number of patients with ILI is obtained.

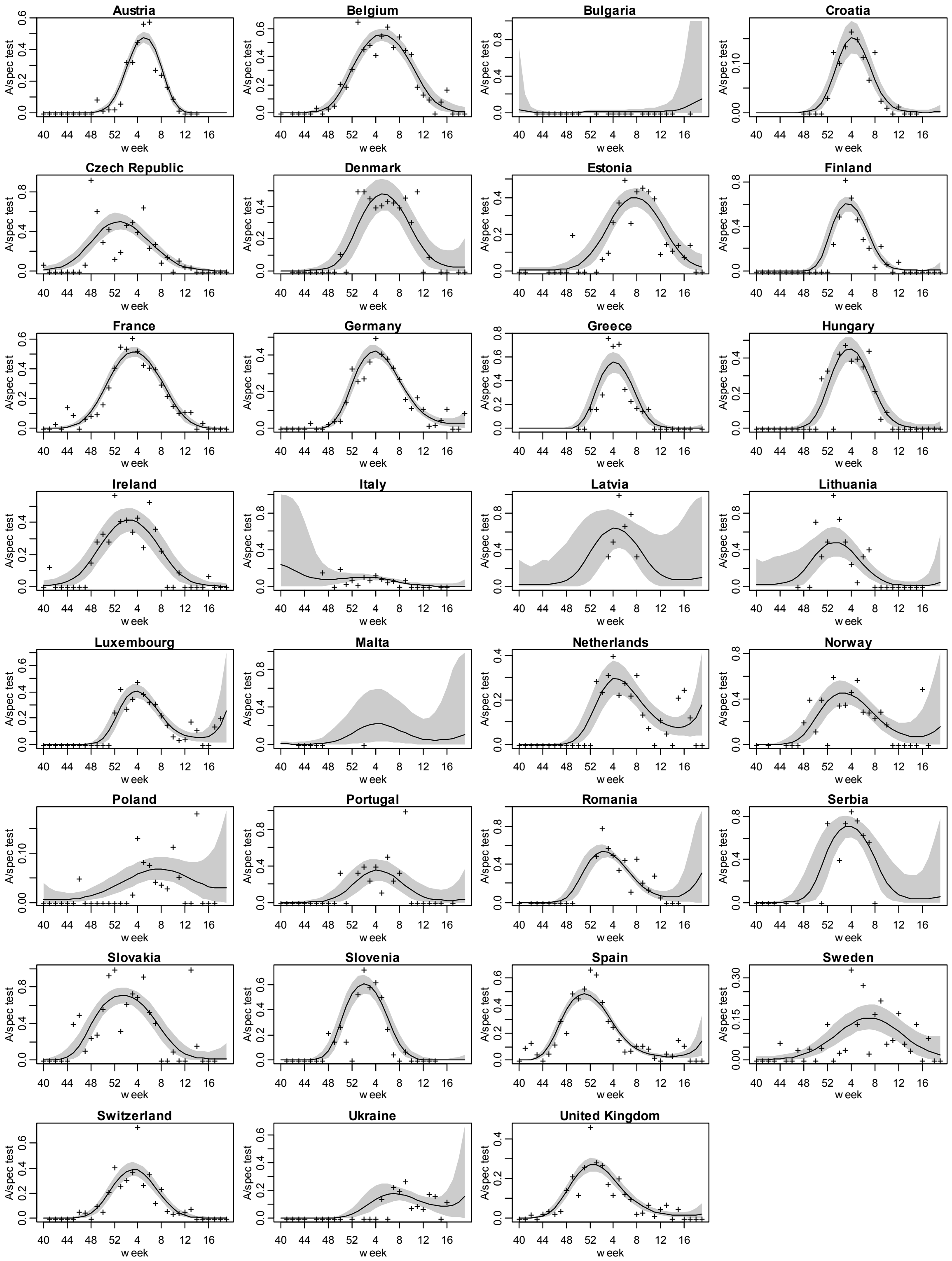

Technical Appendix Figure 2. Fitted curves to the percentage of influenza A virus detections per number of tested specimens from sentinel patients with influenza-like illness. These fractions are more uncertain than those in Figure 1 because of smaller denominators or missing data. Multiplying the fractions of Figure 1, the fractions of Figure 2, and the total population gives the target population.

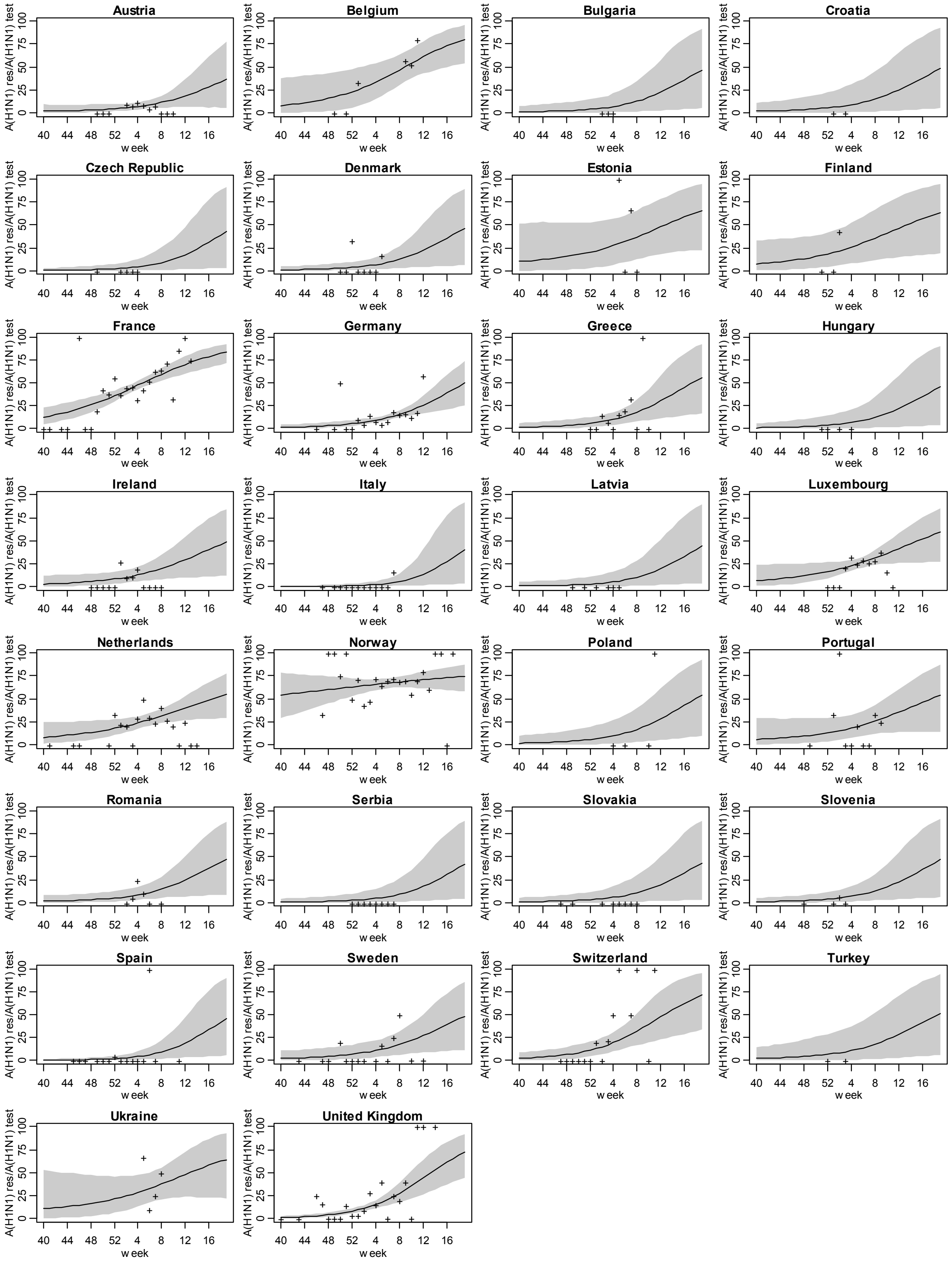

Technical Appendix Figure 3. Fitted curves to the percentage of oseltamivir-resistant influenza viruses A (H1N1) per number of influenza viruses A (H1N1) tested, from sentinel and nonsentinel specimens combined. Countries with only few observations show large uncertainties and curves that are close to the mean. Generally, the prevalence increases during the season.

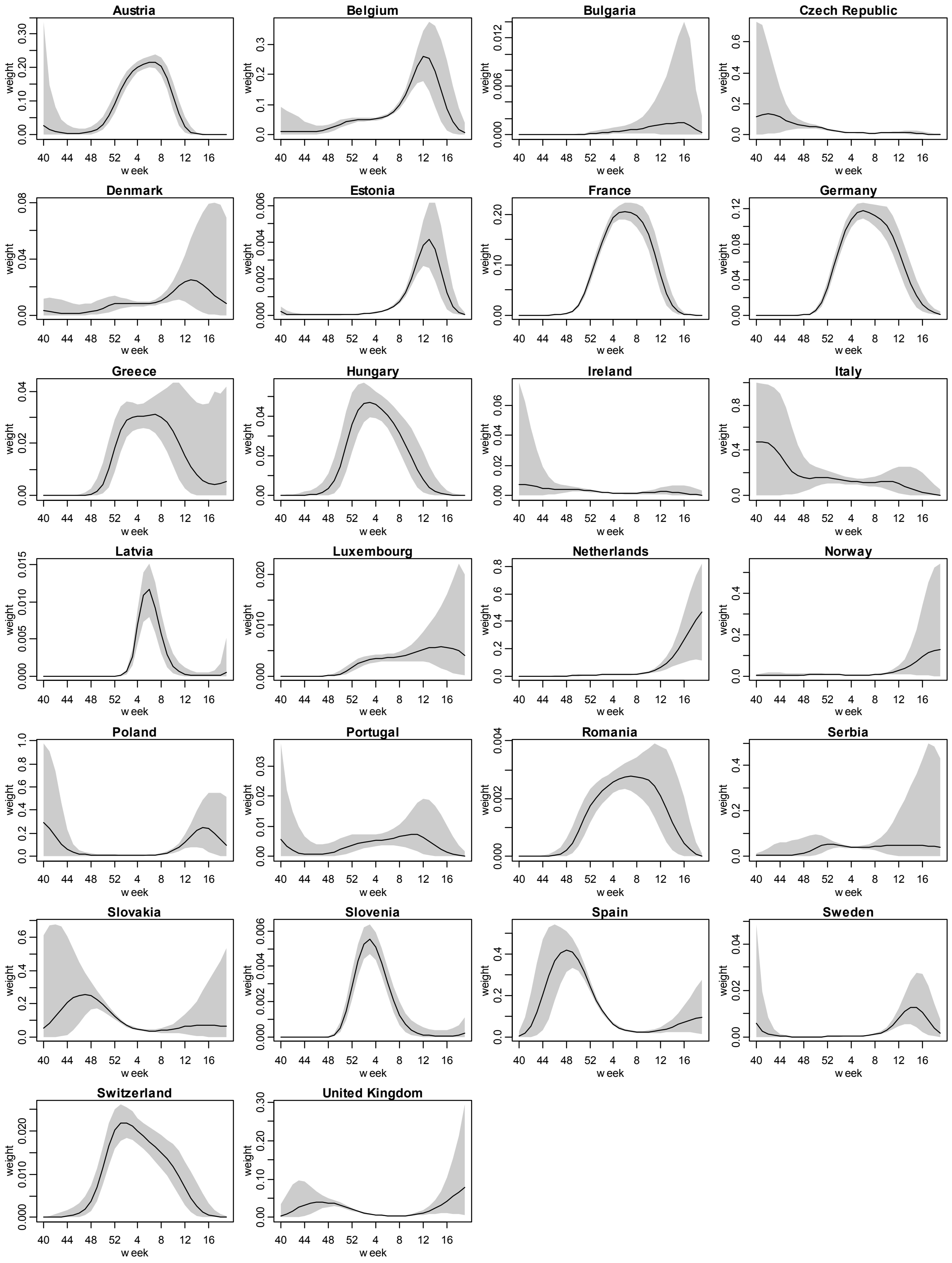

Technical Appendix Figure 4. Relative weights by country, obtained by dividing the target population number by the sum of the target populations for all countries. For any given week, the weights should total 1.

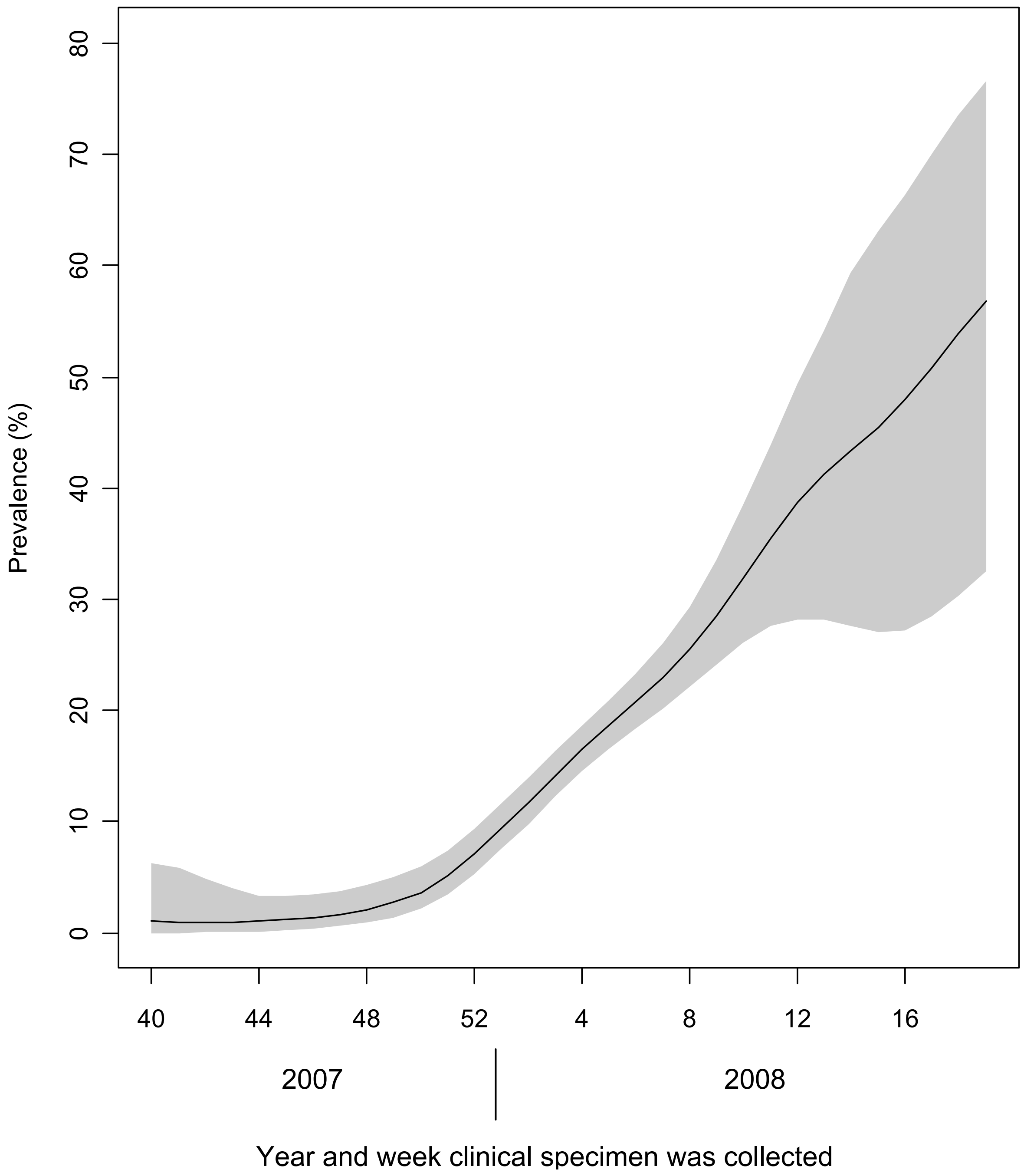

Technical Appendix Figure 5. Prevalence of oseltamivir-resistant influenza viruses A (H1N1) in Europe obtained by multiplying the prevalences of Figure 3 with the weights of Figure 4 and summed over all countries. As in Figure 3 for most of the countries, the European prevalence increases during the season.
